# The Gut‐Brain Connection: How Age, Sex, and APOE ε4 Influence Probiotic Balance in Early Alzheimer's Disease

**DOI:** 10.1002/alz70860_096704

**Published:** 2025-12-23

**Authors:** Warnakulasuriya MADB Fernando, Ralph N Martins, Stephanie R Rainey‐Smith, Hamid R Sohrabi, Samantha Ramachandra, Sithara Udeshini Dissanayaka

**Affiliations:** ^1^ Edith Cowan University, Perth, Western Australia, Australia; ^2^ Australian Alzheimer's Research Foundation, Perth, Western Australia, Australia; ^3^ Co‐operative research Centre (CRC) for Mental Health, Carlton South, VIC, Australia; ^4^ Alzheimer's Research Australia, Perth, Western Australia, Australia; ^5^ KaRa Institute of Neurological Diseases, Sydney, NSW, Australia; ^6^ McCusker Alzheimer's Research Foundation, Perth, Western Australia, Australia; ^7^ School of Medical and Health Sciences, Edith Cowan University, Perth, Western Australia, Australia; ^8^ Sir James McCusker Alzheimer's Disease Research Unit (Hollywood Private Hospital), Perth, Western Australia, Australia; ^9^ Centre of Excellence for Alzheimer's Disease Research and Care, School of Medical and Health Sciences, Edith Cowan University, Joondalup, Western Australia, Australia; ^10^ Macquarie University, Sydney, NSW, Australia; ^11^ Department of Biomedical Sciences, Macquarie University, Macquarie Park, NSW, Australia; ^12^ Centre for Ageing, Cognition and Wellbeing, Macquarie University, North Ryde, NSW, Australia; ^13^ School of Medical and Health Sciences, Edith Cowan University, Joondalup, Western Australia, Australia; ^14^ Edith Cowan University, Joondalup, Western Australia, Australia; ^15^ Centre for Healthy Ageing, Murdoch University, Murdoch, Western Australia, Australia; ^16^ Murdoch University, Perth, Western Australia, Australia; ^17^ Alzheimer's Research Australia, Perth, Western Australia, Australia; ^18^ School of Psychological Science, University of Western Australia, Crawley, Western Australia, Australia; ^19^ Australian Alzheimer's Research Foundation, Nedlands, Western Australia, Australia; ^20^ KaRa Institute of Neurological Diseases, Sydney, Australia; ^21^ Murdoch university, Murdoch, Western Australia, Australia; ^22^ Centre for Healthy Ageing, Health Future Institute, Murdoch University, Perth, Western Australia, Australia; ^23^ Australian Alzheimer's Research Foundation, Perth, Western Australia, Australia; ^24^ Ageing, Cognition and Exercise Research Group, Murdoch, Australia; ^25^ Edith Cowan University, Joondalup, Australia; ^26^ Cooperative Research Centre for Mental Health, Melbourne, Australia

## Abstract

**Background:**

Alzheimer's disease (AD) is a progressive neurodegenerative disorder, with evidence suggesting gut microbiota plays a critical role in its onset and progression. Shifts in probiotic communities during the preclinical phase may influence disease pathways through gut‐brain interactions. This study investigates how age, sex, and APOE ε4 genotype impact probiotic composition and microbial metabolite production in cognitively unimpaired individuals.

**Method:**

Stool samples from 123 participants in the Australian Imaging Biomarkers and Lifestyle (AIBL) study and WA Memory Study (WAMS) were analysed. Participants were grouped by age (<70, ≥70 years), sex, and APOE ε4 carrier status.

Metagenomic sequencing assessed gut microbial composition, focusing on probiotics like Bifidobacterium and Lactobacillus.

Gas‐liquid chromatography measured short‐chain fatty acids (SCFAs), including butyrate, propionate, and acetate.

**Result:**

Gut Microbiota Composition: Dominant bacterial phyla included Actinobacteria, Bacteroidetes, Cyanobacteria, and Firmicutes across all groups.

Age‐Related Changes: Older participants (≥70 years) showed significant declines in Bacteroidetes and Firmicutes, reflecting reduced microbial diversity.

Sex‐Specific Differences: Females had lower Firmicutes levels, reducing butyrate production, essential for inflammation control and brain health.

APOE ε4 Carriers: Older APOE ε4 carriers showed a decline in butyrate‐producing bacteria, particularly Faecalibacterium prausnitzii, leading to reduced butyrate and elevated acetate levels.

Sex and APOE ε4: Female APOE ε4 carriers ≥70 exhibited the most pronounced butyrate decline, indicating increased vulnerability to dysbiosis and inflammation.

Probiotic Alterations: Key probiotics, including Bifidobacterium and Lactobacillus, were significantly reduced in older APOE ε4 carriers.

**Conclusion:**

Age, sex, and APOE ε4 status significantly influence gut microbiota composition and SCFA production at the preclinical stage of AD. Reduced butyrate levels, particularly in older female APOE ε4 carriers, highlight the importance of gut health in mitigating AD risk. These findings suggest targeted probiotic interventions could restore gut balance and delay AD progression.

